# Next Generation Diagnostics in Inherited Arrhythmia Syndromes: A Comparison of Two Approaches

**DOI:** 10.1007/s12265-012-9401-8

**Published:** 2012-09-07

**Authors:** James S. Ware, Shibu John, Angharad M. Roberts, Rachel Buchan, Sungsam Gong, Nicholas S. Peters, David O. Robinson, Anneke Lucassen, Elijah R. Behr, Stuart A. Cook

**Affiliations:** 1 MRC Clinical Sciences Centre, Imperial College London, London, UK; 2 National Heart and Lung Institute, Imperial College London, London, UK; 3 Cardiovascular Biomedical Research Unit, Royal Brompton & Harefield NHS Trust, London, UK; 4 Wessex Regional Genetics Laboratory, Salisbury NHS Foundation Trust, Salisbury, UK; 5 Faculty of Medicine, University of Southampton, Southampton, UK; 6 St George’s University of London, London, UK; 7 Molecular Cardiology, MRC Clinical Sciences Centre, Imperial Centre for Translational and Experimental Medicine, Imperial College London, London, W12 0NN UK

**Keywords:** Inherited cardiac conditions, Next-generation sequencing, Molecular diagnosis, Genetics, Ion channels, Long QT syndrome

## Abstract

Next-generation sequencing (NGS) provides an unprecedented opportunity to assess genetic variation underlying human disease. Here, we compared two NGS approaches for diagnostic sequencing in inherited arrhythmia syndromes. We compared PCR-based target enrichment and long-read sequencing (PCR-LR) with in-solution hybridization-based enrichment and short-read sequencing (Hyb-SR). The PCR-LR assay comprehensively assessed five long-QT genes routinely sequenced in diagnostic laboratories and “hot spots” in RYR2. The Hyb-SR assay targeted 49 genes, including those in the PCR-LR assay. The sensitivity for detection of control variants did not differ between approaches. In both assays, the major limitation was upstream target capture, particular in regions of extreme GC content. These initial experiences with NGS cardiovascular diagnostics achieved up to 89 % sensitivity at a fraction of current costs. In the next iteration of these assays we anticipate sensitivity above 97 % for all LQT genes. NGS assays will soon replace conventional sequencing for LQT diagnostics and molecular pathology.

**Electronic supplementary material:**

The online version of this article (doi:10.1007/s12265-012-9401-8) contains supplementary material, which is available to authorized users.

## Introduction

Genetic subtypes of inherited cardiac conditions (ICCs), such as long QT (LQT) syndrome, are associated with distinct patterns of risk and a molecular diagnosis can be used to direct clinical management [1–5] and permit cascade screening in families, which is more effective than clinical screening alone [6]. However, ICCs are genetically heterogeneous [7] and conventional sequencing of ICC genes is expensive, time-consuming and rarely and inequitably applied in clinical practise, notwithstanding published guidelines [6].

The maturation of recently developed next generation sequencing (NGS) technologies provides unprecedented sequencing capacity at dramatically lower cost, and NGS has been implemented by some healthcare providers in the US and Europe for clinical diagnostics of ICCs (e.g., GeneDx, USA; Sistemas Genómicos, Spain; Oxford Molecular Genetics, UK). To date there have been no published studies that address the specific challenges of applying NGS technologies to inherited arrhythmia syndromes that include LQT syndrome, Brugada syndrome (BrS) and catecholaminergic polymorphic VT (CPVT).

A major challenge associated with targeted NGS is efficient and specific enrichment of disease genes prior to sequencing as, unlike Sanger sequencing [8], NGS approaches have no intrinsic target specificity [9]. To achieve target enrichment prior to NGS, DNA libraries are most commonly enriched for sequences of interest by PCR- or hybridization-based methods. PCR-based methods are typically multiplexed or parallelised in order to produce amplicons on a scale appropriate for NGS. Mature parallel approaches may separate PCR reactions in microdroplets (e.g., RDT 1000, RainDance Technologies) or microfluidic chips (e.g., Access Array, Fluidigm) before pooling amplicons. More recently, kits for pooled ultrahigh-multiplex PCR have been released (e.g., Ion Ampliseq, Life Technologies). The first generation Access Array is appropriate for targeted resequencing of small (<25,000 bp) regions: a library of 48 amplicons can be prepared from each of 48 samples in parallel (2,304 independent PCR reactions) in less than a day, and with little hands-on time [10]. Hybridisation approaches use specific labelled oligonucleotide baits to separate DNA containing sequences of interest from background, either in solution or bound to a microarray chip. Examples include SeqCap EZ Library (in-solution) and Sequence Capture Arrays (both Roche NimbleGen), TruSeq in-solution (Illumina) and the SureSelect in-solution system (Agilent Technologies). These provide off-the-shelf solutions to capture the whole exome, and customized versions for user-defined targets, the SureSelect custom system having a capacity of up to 6 Mb [11, 12].

NGS platforms are often divided into two categories on the basis of the length of sequence reads that they produce. Perhaps the most established long-read platform is the 454 GS FLX (454 Life Sciences, Roche) that produces reads of up to 1,000 bp. The smaller, table-top GS junior platform (also Roche) produces 400-bp reads and a total sequencing output of ~40 Mbp (10-h run). Short-read platforms, such as the Illumina Genome Analyzer II, Illumina HiSeq 2000 (Illumina), Applied Biosystems SOLiD v4 and SOLiD 5500 series (Life Technologies), typically have a significantly higher throughput than the long-read platforms, making them more suitable for sequencing a larger number of genes.

Here we present a comparison of two distinct approaches for NGS high-throughput diagnostics of genes causing inherited arrhythmia syndromes: PCR-based target enrichment using the Access Array followed by long-read sequencing on the GS junior (PCR-LR) and enrichment by SureSelect in-solution hybridization with short-read SOLiD v4 sequencing (Hyb-SR).

## Methods

### Sample Selection

The Hammersmith and Queen Charlotte’s and Chelsea Research Ethics Committee approved the study. DNA was obtained from subjects who had given written informed consent and was provided in accordance with Human Tissue Act, UK guidelines. DNA was extracted using standard automated approaches and quality and quantity was assessed by agarose gel electrophoresis and fluorometry (Qubit, Life Technologies).

A total of 48 patient samples were sequenced. Of these, 33 were sequenced using both approaches, and 15 using one or other approach (PCR-LR *n* = 12, Hyb-SR *n* = 3) (Table S1). Samples included a number of positive controls with variants previously identified by Sanger sequencing. Nineteen positive control variants in KCNQ1, KCNH2 and SCN5A were sequenced using both assays for direct comparison (Table 1).

**Table 1:** Detection of positive control variants in samples sequenced on both platforms

Variant type	Disease	Gene	Variant	Sample ID	SOLiD PSS	454 PSS	SOLiD GATK	454 GATK
SNP	LQT	SCN5A	c.6016C > G	02	Yes	Yes	Yes	Yes
SNP	LQT	KCNQ1	c.859G > A	02	Yes	Yes	Yes	Yes
SNP	LQT	KCNH2	c.1744C > T	02	Yes	Yes	Yes	No
SNP	LQT	KCNQ1	c.1697C > T	04	Yes	Yes	Yes	Yes
Indel	LQT	KCNH2	c.1152delG	05	Yes	No	No	–
SNP	LQT	KCNH2	c.1926C > G	06	Yes	No	No	No
Indel	LQT	KCNH2	c.1152delG	08	Yes	No	No	–
SNP	LQT	KCNQ1	c.965C > T	10	Yes	Yes	Yes	Yes
Indel	LQT	KCNQ1	c.1486_1487delCT	11	Yes	Yes	Yes	–
Indel	LQT	KCNH2	c.2775dupG	12	No	No	No	–
Indel	LQT	KCNH2	c.1916_1918delTCT	13	Yes	Yes	Yes	–
SNP	BrS	SCN5A	c.2236G > A	14	Yes	Yes	Yes	Yes
Indel	LQT	KCNQ1	c.796delC	15	Yes	No	Yes	–
SNP	LQT	KCNQ1	c.569G > A	16	Yes	Yes	Yes	No
SNP	LQT	KCNQ1	c.1702G > A	17	Yes	Yes	Yes	No
SNP	LQT	KCNQ1	c.569G > A	18	No	Yes	No	Yes
SNP	BrS	SCN5A	c.6010T > C	27	Yes	Yes	No	No
SNP	LQT	KCNQ1	c.1075C > T	31	Yes	Yes	Yes	Yes
SNP	LQT	KCNQ1	c.1781G > A	32	Yes	Yes	No	Yes

Sensitivity (%)			All variants		89	74	63	n/a
			SNPs		92	92	69	62
			Indels		83	33	50	n/a

There is no significant difference in sensitivity between platforms (SOLiD PSS v 454 PSS: *p* = 0.375). Comparison of software packages within platform (including additional control variants sequenced on only one platform, listed in Table S1) demonstrates that PSS is more sensitive (454, *p* = 0.031; SOLiD, *p* = 0.031)

Reference sequences: KCNQ1 = ENST00000155840, KCNH2 = ENST00000262186, SCN5A = ENST00000333535. See Methods for variant calling parameters for each approach

*PSS* platform specific software, *GATK* Genome Analysis Toolkit, *Indel* insertion or deletion, *LQT* long QT syndrome, *BrS* Brugada syndrome, *n/a* not applicable

### Assay Designs

#### PCR-LR

Fluidigm’s commercial design and validation service was used to design 96 amplicons targeting five LQT genes (KCNQ1, KCNH2, SCN5A, KCNE1 and KCNE2) for which clinical testing is currently available in the UK, together with a subset of exons from RYR2 based on the prioritisation schema proposed by Medeiros-Domingo et al. [13]. Amplicons ranged from 248 to 600 bp in length, and extended a variable distance into adjacent introns to cover exon/intron boundaries. The 96 amplicons covered 42,023 bp of sequence, of which 16,123 bp was our core protein-coding target, and the remainder adjacent intronic sequence and UTR.

#### Hyb-SR

**Fig. 1:**
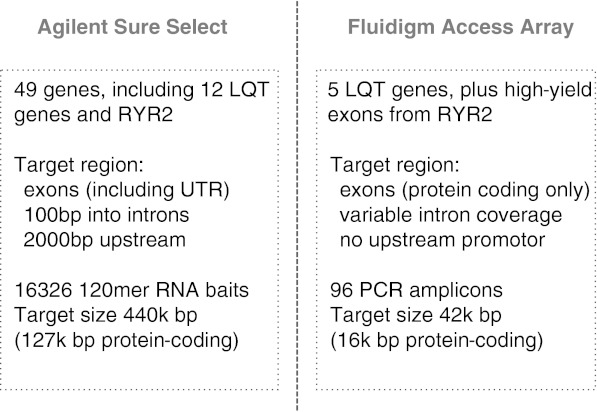
Summary of target selection designs for the two target enrichment strategies. *LQT* long QT syndrome, *bp* base pairs

RNA baits were designed for 49 inherited arrhythmia genes (Table S2) using Agilent’s eArray platform (https://earray.chem.agilent.com/earray). Baits targeted all exons of all Ensembl transcripts of these genes, downloaded from Ensemble version 54 [14], including UTRs, a 100-bp overhang into adjacent introns, and 2 kb of sequence upstream of the earliest transcription start site. A total of 16,326 unique 120 mer RNA baits were generated with standard eArray parameters other than five-fold tiling across the target regions (eArray parameters: sequencing protocol = end-sequencing, tiling frequency = 5× for exons and adjacent intronic overlap, 2× for 2 kb upstream sequence, bait length = 120, standard repeats = off, avoid overlap = 20, layout strategy = centred), covering a target region of 448,412 bp, including 126,638 protein-coding bases. An overview is provided in Fig. 1.

### Library Preparation and Sequencing

The workflow is summarised in Fig. S1, and calculations of anticipated assay capacity are given in Table S3.

#### PCR-LR

A total of 96 amplicons were prepared from 45 samples in two 48.48 Access Array IFC chips according to the manufacturer’s standard protocol. In brief, 50 ng (1 μl) of each sample was combined with barcode library and PCR mastermix (Roche FastStart High Fidelity PCR System), and transferred to the primed Access Array chip. Forward and reverse tagged target-specific primers for each amplicon were added, and target regions were amplified with incorporation of barcodes and sequencing adaptors in a single nested PCR. Pooled amplicons from each sample were harvested, and 2 μl of product per sample from each chip was pooled, purified and quantified. The pooled library was prepared for sequencing on the GS junior using the manufacturer’s protocol. Emulsion PCR (ePCR) was carried out using a ratio of 0.8 copies per bead, and 500,000–2,000,000 beads were recovered from ePCR and sequenced in one run.

#### Hyb-SR

A total of 36 samples were enriched and barcoded using the SureSelect system according to the manufacturer’s standard protocols, in batches of eight (low multiplex) and 24 (high multiplex). First, 3 μg of DNA in 120 μl of low TE was sheared, end-repaired and ligated with sequencing adaptors. Next, 200- to 250-bp fragments were selected using agarose gel electrophoresis, prior to nick-translation and amplification. Then, 500 ng of DNA was then incubated with target-specific biotinylated RNA baits for 24 h, and the target DNA captured using streptavidin-coated magnetic beads. Libraries were quantified by qPCR and pooled. Following ePCR, libraries were sequenced on the SOLiD v4 using paired-end sequencing.

### Data Analysis

Two analysis pipelines were compared for each NGS platform. In each case the manufacturer’s proprietary platform-specific variant-calling software was compared against the freely available and widely used Genome-Analysis Toolkit (GATK, v1.0.5232) [15].

#### PCR-LR

GS Amplicon Variant Analyser (AVA, version 2.5p1) was used as an integrated system for read trimming, de-multiplexing and variant calling. This software provides limited user-accessible data on read quality and coverage, so for comparability against the SOLiD data a custom pipeline was also used. In this pipeline reads were de-multiplexed, trimmed, and converted to FASTQ format using SffTools (454 Sequencing System Software, v2.5p1) and sff2fastq (http://github.com/indraniel/sff2fastq). Short and long reads (cut-off = 100 bp) were aligned separately to the human reference genome (hg19) using BWA-short and BWA-SW, respectively [16, 17] and aligned reads recombined. Alignment metrics were calculated with Picard v1.37 (http://picard.sourceforge.net). Coverage data were obtained using SAMtools v0.1.12-10 (r896) [18], Picard, BedTools [19] and the GATK Callable Loci Walker [20]. Bases covered by at least four reads with a mapping quality ≥20 and base quality ≥10 were denoted “callable”, i.e., adequately covered for variant calling with recommended GATK parameters. High quality aligned reads were passed to GATK for SNP calling (as GATK does not support indel calling on 454 data) using recommended parameters and filters (min_base_quality_score = 10; min_mapping_quality_score = 20; stand_call_conf = 10.0; stand_emit_conf = 30.0; minIndelCnt = 4. Downstream filters: QUAL < 30, QD < 5, HRun > 5; DP < 4). Putative variants identified by AVA were accepted if present on both strands with total coverage of at least four reads.

#### Hyb-SR

SOLiD reads were de-multiplexed and aligned in colour space using SOLiD BioScope v1.3. Duplicate reads were marked using Picard, and alignment and coverage metrics obtained as previously described for the GS junior data. Variant calling was carried out using GATK and BioScope using recommended parameters (GATK parameters as above. Lifescope: het/hom.min.coverage = 3; het.min.allele.ratio = 0.15; reads.min.mapping.qv = 20; het/hom.min.nonref.color.qv = 10; call.stringency = medium; small.indel.min.num.evid = 4; small.indel.min.best.mapping.quality = 20. Downstream filter: diBayes *p* < 0.05).

Target enrichment factor was calculated as ${\text{Enrichment}}\,{\text{Factor}} = \frac{{{\text{Reads}}\,{\text{on}}\,{\text{target/Total}}\,{\text{mapped}}\,{\text{reads}}}}{{{\text{Target}}\,{\text{size/Genome}}\,{\text{size}}}}$. Here, “target” refers to all protein-coding bases only, though amplicons/baits were designed to capture adjacent regions.

For comparability, the depth of coverage, proportion of bases meeting variant calling criteria, and the evenness of coverage were calculated for the protein-coding portions of the six genes common to both assays. The same metrics were also calculated for all genes in the Hyb-SR assay. Evenness was calculated according to the method described by Mokry et al. [21], implemented with the R statistical package (http://www.r-project.org/) using a custom script. This yields a score in the range 0–1, with 1 indicating perfectly uniform coverage. The correlation between sequencing depth and callable bases was assessed using Spearman’s rho, implemented in R (*stats* package, version 2.13.1).

Variants were functionally annotated using the Ensembl API (version 63) [22] and HGMD Professional version 2011.1 [23]. The number of positive-control variants detected by each platform and each analysis pipeline was compared using the McNemar exact test, implemented with the *exact2x2* package in R [24].

## Results

### Target Enrichment and Sequencing Metrics

The sequencing output and enrichment statistics are summarized in Table S4. PCR-LR had high target specificity, with 89 % of uniquely mapped reads on target (mean enrichment factor = 170,900). The reads that did not map were almost exclusively short DNA fragments: 22 % of reads were short (<100 bp), of which <1 % mapped, whereas 99.97 % of longer reads mapped. For Hyb-SR, up to 25 % of mapped reads were on target, which is as expected for in solution hybridization with a relatively small target size. [12] Of these on target reads 49 % were flagged as duplicates prior to variant calling at low sequencing depth, rising to 84 % with increased sequencing. Mean enrichment factor was 3,505 at high multiplex, and 5,864 at low multiplex.

### Coverage of Targeted Genes

**Fig. 2:**
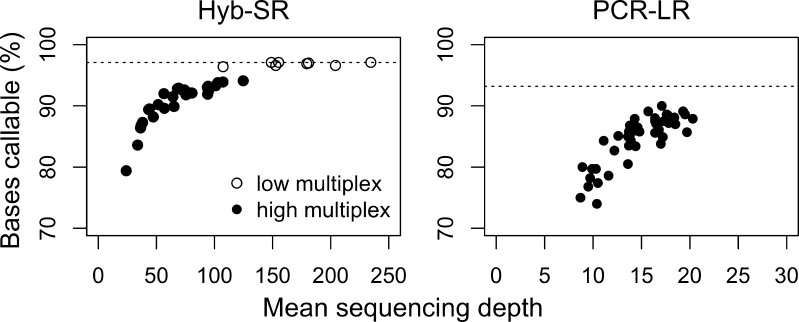
For a given target enrichment design, the percentage of bases reaching variant-calling criteria increases with increasing sequencing depth. For each sample, the percentage of target bases callable is plotted against the mean sequencing depth achieved for that sample. The Hyb-SR reached saturation when run with low multiplex, suggesting that a further increase in sequencing depth would not improve coverage. PCR-LR has not reached saturation: the *dotted line* shows maximum achievable coverage with a simulated sample generated by pooling reads from all samples, equivalent to ×660 depth

We compared assay performance using the six genes sequenced using both approaches (KCNQ1, KCNH2, SCN5A, KCNE1, KCNE2 and RYR2), and determined what proportion of each gene was covered adequately to identify variants (“callable”). Target capture and sequencing depth both contribute to determine whether a base is callable, so to dissect these the effect of sequencing depth was first investigated. We demonstrated a strong correlation between bases callable and mean sequencing depth across samples (Hyb-SR, *ρ* = 0.95, *p* < 2.2 × 10^−16^; PCR-LR, *ρ* = 0.81, *p* < 2.2 × 10^−16^), but with better performance of the PCR-LR at lower coverage (Fig. 2). In each case the relationship is approximately linear at low sequencing depth, and then approaches a plateau when all of the bases captured by upstream enrichment are covered at this minimum depth. Hyb-SR at low multiplex has reached this plateau, and therefore represents the best possible coverage achievable by this target enrichment design. Hyb-SR at high multiplex has not yet reached the plateau: in clinical application mean sequencing depth would need to be increased to 250–300× ensure maximum coverage. As PCR-LR is not sequenced at sufficient depth to eliminate this factor, a high coverage virtual sample was generated by pooling reads from many samples (~90 k reads, yielding 663× coverage). This pooled sample has been used to compare target capture under optimum conditions.

The depth of coverage of the target region was higher using Hyb-SR, but the regions of these six genes sequenced to sufficient depth to confidently identify variants were similar for both approaches (Table 2, six genes, both assays; Table S2, 49 genes in Hyb-SR assay). Of note, the performance of the Hyb-SR across the six LQT genes was not as good as the average performance of Hyb-SR across all 49 genes in this comprehensive assay (Table 3), perhaps reflecting the high GC content of some LQT genes.

**Table 2:** Percentage of bases covered sufficiently for variant calling by gene, for each assay (median across samples). For Hyb-SR, results are shown from a low multiplex run with high sequencing depth, approximating to optimal performance for this assay. The PCR-LR run was relatively under-sequenced, so increased sequencing depth was simulated by pooling reads from many samples. PCR-LR would yield better coverage with increased sequencing depth (e.g., fewer multiplexed samples)

Gene	Syndrome	Hyb-SR (low multiplex)	PCR-LR	PCR-LR pooled (high-coverage)
KCNQ1	LQT1	83.4	80.9	80.9
KCNH2	LQT2	78.2	80.8	89.2
SCN5A	LQT3	99.9	89.2	97.8
KCNE1	LQT5	100.0	100.0	100.0
KCNE2	LQT6	100.0	100.0	100.0
^a^RYR2	CPVT	100.0	87.8	89.2

^a^With respect to the RYR2 exons targeted by both assays. Performance across the whole RYR2 gene for the Hyb-SR is shown in Table S2

**Table 3:** Target enrichment: coverage of targeted bases

Assay	Bases covered (%)				Mean (median) sequencing depth	Evenness
	≥1×	≥5×	≥10×	Callable^a^		
Performance across shared target (6 genes; 16,123 bp)						
Hyb-SR low multiplex	95.6	93.8	92.6	93.3	163 (177)	0.79
Hyb-SR high multiplex	94.0	90.0	87.4	85.4	55.8 (54.5)	0.72
PCR-LR	90.5	82.5	68.4	85.6	14.4 (14)	0.72
PCR-LR pooled (simulated high coverage)	93.2	93.2	93.2	93.2	663 (687)	0.77
Performance across whole target (49 genes; 126,638 bp)						
Hyb-SR low multiplex	98.3	97.3	96.6	97.0	167 (176)	0.82
Hyb-SR high multiplex	97.4	95.2	93.5	92.0	68.6 (73)	0.79

Data is shown for two Hyb-SR runs (at low and high multiplexes), PCR-LR, and a simulated high coverage PCR-LR sample. Hyb-SR at low multiplex and PCR-LR pooled give an indication of the maximum performance of each target enrichment design with sufficiently deep sequencing

^a^Callable = percentage of bases meeting variant calling criteria, assessed using GATK callable loci walker

**Fig. 3:**
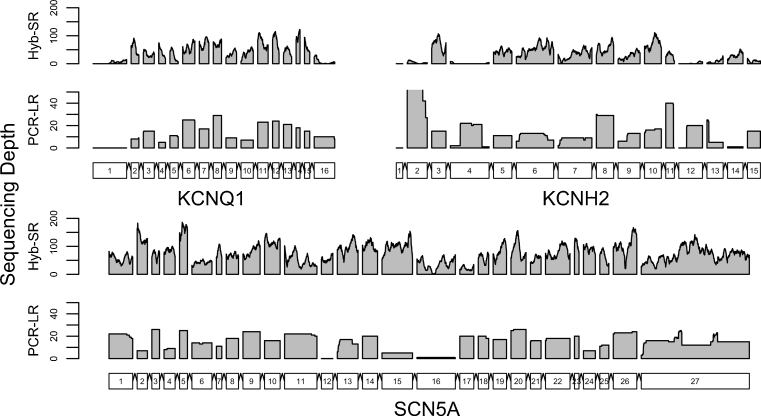
Coverage of the three genes most commonly causing long QT syndrome. Sequencing depth is plotted base by base across the protein-coding portions of three genes for a single sample sequenced on both platforms. Coverage varies widely for the Hyb-SR approach. PCR-LR yields more even coverage within amplicons, but there remains significant inter-amplicon variability. The first exons of KCNQ1 and KCNH2 are poorly captured by both techniques. The proportion covered sufficiently for variant calling ranges from 78 % (KCNH2, Hyb-SR) to 100 %

**Fig. 4:**
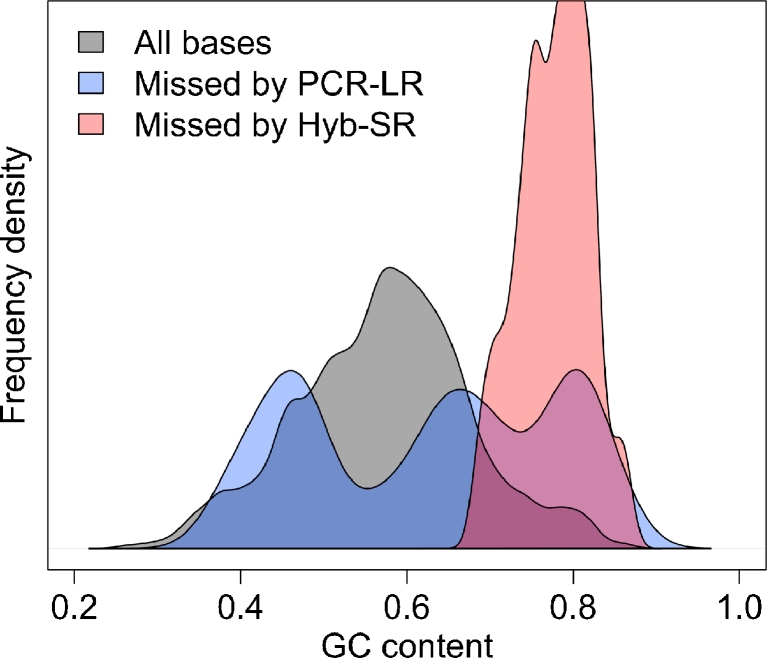
Target enrichment is strongly dependent on GC content. The distribution of GC content for the target region is shown, together with the GC content of bases consistently missed across all samples for each platform. Regions missed by Hyb-SR have a high GC content, while regions missed by PCR-LR may have a GC content at either extreme

Visual representation of the coverage of the three principal LQT genes (Fig. 3) revealed the variable capture performance across the genes. Regions missed tended to have extreme GC content (Fig. 4), with many of these regions common to both platforms. PCR-LR is sensitive to both extremes of GC, while Hyb-SR appears robust to low GC content. A previous study has shown that this is a distinctive feature of the SureSelect system, which outperforms other hybridisation-based enrichment systems in regions of low GC content [25]. Qualitatively, Hyb-SR yields highly variable coverage across the target, while PCR-LR yields even coverage within amplicons, but significant inter-amplicon variability and some areas of high coverage where two amplicons overlap. Quantitatively, PCR-LR coverage is no more even than Hyb-SR coverage.

### Variant Detection

Using the proprietary variant-calling pipeline for each platform there is no significant difference in detection of 19 known variants between platforms (Table 1; Hyb-SR 89 %, PCR-LR 74 %, *p* = 0.375). In general, variants that were not detected were in areas of poor coverage, suggesting that they were not captured adequately by the target-enrichment strategies, not sequenced adequately (due to emulsion PCR or sequencing biases), or not uniquely aligned during sequence alignment. The only variant to be missed by both variant-calling approaches on both platforms (KCNH2 c.2775dupG) fell in a region with 79 % GC yielding no coverage on PCR-LR and two reads on Hyb-SR. Other missed variants were largely attributable to either low/no coverage, or coverage-related variant-calling filters in GATK (e.g., Quality by Depth). It is notable that the variants missed were in regions of higher GC content than those consistently detected (GC 65 ± 3 % vs. 59 ± 2 %, *p* = 0.014).

For each approach we compared GATK against platform-specific software (BioScope, SOLiD; AVA, GS junior). In both cases, there were no positive control variants that were detected by GATK and missed by the platform-specific software, while a modest number were detected by platform-specific software and missed by GATK (Table S5). Platform-specific software is statistically more sensitive than GATK (454, *p* = 0.031; SOLiD, *p* = 0.031).

### Cost and Time Comparisons

Comparative costs for consumables alone are shown in Table 4. Both NGS approaches represent a significant cost saving over conventional sequencing, although these figures do not include capital, maintenance, or informatics costs for either NGS or conventional capillary sequencing. A single high-throughput capillary sequencer could, in theory, sequence five LQT genes (66 amplicons) in 17 samples in 1 day, with significant additional time required for upstream PCR and sample preparation. PCR-LR takes 2 days for target enrichment and sequencing of 48 samples. Time estimates for Hyb-SR depend on whether upstream automation is used, with the sequencing itself taking up to 2 weeks.

**Table 4:** Consumables costs for target enrichment and sequencing technologies

Technology	Cost per sample
Conventional Sanger sequencing^a^ (5 genes)	£475 ($736, €594)

Access Array target enrichment^b^ (5½ genes)	£10
GS junior sequencing^c^	£25
PCR-LR Total	£35 ($54, €44)

SureSelect target enrichment^d^ (49 genes)	£90
SOLiD v4 sequencing^e^	£35
Hyb-SR total	£125 ($194, €156)

^a^PCR and bidirectional sequencing of 66 amplicons (5 LQT genes), at £3.60 per amplicon

^b^Price excludes outsourcing of design and validation of primers (=£3450 for 48 amplicons)

^c^Forty-eight samples on one GS junior run

^d^Includes design cost, and assumes bulk purchase of 1,000 captures

^e^Thirty-two samples in one quarter of a SOLiD slide, 50 + 35 paired-end sequencing

## Discussion

NGS is a mature technology for clinical diagnostics and promises comprehensive genetic assessment at low cost. While the initial LQT assays described here will require optimisation prior to clinical application, some targeted genes are already fully sequenced (Table 2). We point out that while conventional sequencing (or mutation scanning) has a notional sensitivity for the detection of SNPs and small indels approaching 100 %, this is only the case if sequencing is applied to all exons of all genes. In practice, this is seldom the case. Most often analysis is limited to a subset of disease genes, or a limited number of exons of a gene (http://www.genetests.org/). It remains unknown, for example, how the overall diagnostic yield of a test that detects 90 % of variants in 50 genes might compare with a test detecting 99 % of variants in five genes. At the same time, background noise increases as the size of the diagnostic gene panel increases, making the discrimination of pathogenic and benign variants more challenging.

Conventional sequencing is not the gold standard for detection of other forms of pathogenic variation, and conventional diagnostics is evolving to incorporate new technologies, such as multiplex ligation-dependent probe amplification for copy number variation detection. Methods to identify large structural variants and copy number variants directly from NGS are now available [26]: these are more likely to be applicable to hyridisation-based targeted enrichment.

### Improving Assay Performance

To improve the performance of the NGS assays described here target capture clearly requires optimisation, though initial results are comparable to those previously reported for a cardiomyopathy screen based on on-array hybridisation and SOLiD sequencing [27] (91 % coverage at 10×, enrichment factor 2,169). Sub-optimal target enrichment may have arisen at several stages. A minority of regions were excluded from the design due to strict amplicon or bait design parameters (e.g., no Access Array amplicon was successfully designed for KCNQ1 exon 1; Fig. 3). Although not quantifiable, failure in bait or primer design during production may also have occurred. Variability in assay performance is likely primarily a function of efficiency of hybridisation and/or PCR, which are known to be sensitive to factors such as GC content (Fig. 4) [12]. Indeed, we have observed some improvement in the Access Array performance using an alternative GC-robust PCR approach (data not shown).

Assay performance could be improved by increasing sequencing depth per sample, pushing borderline regions above variant calling threshold (Fig. 2). However, this is inefficient if coverage is uneven, as sequencing of efficiently captured regions also increases. Redistribution of sequencing through more even capture would be preferable. PCR-based enrichment is said to produce even coverage [10], but our data show no advantage over hybridisation-based enrichment. The majority of bases not callable in the PCR-LR assay reflected no coverage, rather than low coverage, while the converse is true for Hyb-SR. This suggests that assay rebalancing through bait re-distribution may be most relevant for hybridisation-based enrichment, while amplicon performance tends towards all-or-none for PCR.

### Variant Calling and Detection

We found that platform-specific proprietary variant calling software has a higher sensitivity than GATK, a widely accepted open-source package. Although GATK is platform-independent, it has developed alongside projects such as the 1000 genome project [28], for which most data has come from the Illumina platform and at low coverage. The Bioscope software has been written specifically to take advantage of the colour-space base encoding on the SOLiD. The AVA software, whilst more sensitive than GATK, does not produce quality values for variant calls, is less transparent and has few customisable parameters. Overall, for an initial experiment, both platforms performed appropriately in detecting previously identified causative mutations (Table 1) with a trend to better performance with Hyb-SR.

Although this study was not powered to compare the detection of different variant classes (insertions, deletions and duplications vs. SNPs), it has previously been noted that insertions and deletions are more difficult to detect in NGS data [9, 27]. We observed that Hyb-SR detects six out of seven such variants, while PCR-LR detects two of six variants (Table 1 and Table S1). This warrants further investigation, particularly given the known sensitivity of 454 sequencing to homopolymer regions [9]. The SureSelect system has previously been reported to be more sensitive to small indels at low coverage than other hybridisation-based target-enrichment systems [25].

The Hyb-SR assay is comprehensive, including genes that are rare causes of LQT (not normally tested clinically) and genes for other arrhythmia syndromes. Alongside our core LQT/BrS comparator samples for the study we sequenced a number of additional samples with other arrhythmia phenotypes to explore this. Two positive control variants from patients with arrhythmogenic right ventricular cardiomyopathy were included, and both correctly identified (Table S1). Additional findings included a novel SNP in an essential splice donor site in ANK2 in a patient with otherwise unexplained LQT syndrome (ENST00000357077.4:c.1485 + 2T > C); a non-synonymous SNP in ANK2 that has been previously reported to cause LQT [29], now found in a molecular autopsy sample from a sudden unexplained death victim (ENST00000357077.4:c.10708G > A; ENSP00000349588.4:p.Glu3570Lys); and a novel variant at an essential splice site in RYR2 in a patient with unexplained ARVC (ENST00000366574.2:c.10725 + 1G > T). Although appropriate caution is required when interpreting sequencing data from large panel of genes, these illustrate the potential benefits of a single comprehensive assay.

### Assigning Pathogenicity to Variants

The major challenge in genetic diagnosis is distinguishing between pathogenic variants and benign rare variants. Although projects such as the 1000 Genomes project [28] and UK10K (http://www.uk10k.org/) will improve our knowledge of common and less common variants, many rare variants will not be catalogued. Moreover, variants identified in these projects cannot all be assumed to be benign, as cohorts with incomplete phenotypic information may include some patients carrying unrecognised disease-causing variants, and variants that are insufficient to cause disease in isolation may still contribute to oligogenic inherited disease. One solution is to sequence large multi-racial control cohorts that have very accurate cardiovascular phenotypes — a prospect that is both achievable and affordable using the assays described here.

## Summary

Table 5 gives a comparative overview of the two approaches. PCR-LR has the advantages of low cost, rapid turnaround, and relative ease of use. Although our assay will require at least one cycle of iterative improvement, we anticipate over 97 % of bases will be callable for all LQT genes in our next design (based on our experience in optimisation of other NGS assays). In addition, Fluidigm’s recent PCR multiplexing protocol for the Access Array [30] markedly increases assay capacity and this combined with the higher throughput desktop sequencers (MiSeq, Illumina; Ion Torrent, Life Technologies) promises a rapid and comprehensive sudden death assay.

**Table 5:** A comparative overview of the two approaches assessed in this study

	PCR-LR	Hyb-SR
Assay capacity	Modest (~ 24 kb)	Comprehensive (~6 Mb for custom designs)
Target enrichment cost	Low (~£10 per sample)	Higher (~£100 per sample)
Sequencing cost	Hyb-SR cheaper per unit of sequencing, but cost per sample comparable given PCR-LR likely to be used for more focused assay	
Turnaround time	~2 days	~2 weeks
	User-friendly automated target-enrichment	More complex library preparation. Automation available
Technical considerations	High specificity/enrichment factor	Lower specificity/enrichment factor
	Sensitive to extremes of GC	Sensitive to high GC, relatively robust to low GC
	Low sequencing depth required for given coverage	Higher sequencing depth required
	Platform-specific software less transparent	Platform-specific software easily integrates with other bioinformatic packages
	Indel calling not available in GATK	GATK a fully featured alternative
	PCR amplification prevents CNV detection	CNV detection likely to become available

*Hyb-SR* hybridisation-based target enrichment (SureSelect) and short-read sequencing (SOLiD), *PCR-LR* PCR-based target enrichment (Access Array) and long-read sequencing (454 GS junior)

The Hyb-SR assay we developed is cheaper than Sanger sequencing, although more expensive and time consuming than PCR-LR. The major advantage of the Hyb-SR assay is its capacity, with a much more comprehensive panel of potentially causative genes. There is also the potential for substantial economies of scale with Hyb-PCR as a single assay covering genes for many (or all) ICCs could be run as a single test with very high throughput. By contrast, PCR-LR on the GS Junior platform would require a separate assay for each syndrome, each requiring optimization and validation.

This study also illustrates that target capture performance varies for different genes, and some LQT genes are difficult to capture. Sensitivity and specificity vary with platform and software choices. Early adopters of NGS diagnostics must ensure that measures of sensitivity for each gene are clearly reported in both advertising and genetic test reports.

The challenges of interpreting variants of unknown significance are substantial, but deep sequencing of well-phenotyped normal cohorts using these comprehensive high-throughput assays will powerfully inform our interpretation. In a short time, NGS will transform the genetic testing strategy for LQT, sudden arrhythmic death and other molecular pathologies and change the landscape of genomic cardiovascular medicine beyond recognition.

## Electronic supplementary material

Below is the link to the electronic supplementary material.

ESM 1 Electronic supplementary material (DOCX 326 kb)
