# Supplementary material for: Next Generation Diagnostics in Inherited Arrhythmia Syndromes: A Comparison of Two Approaches
Source: J Cardiovasc Transl Res. 2012 Sep 7;6(1):94–103. doi: 10.1007/s12265-012-9401-8 (PMC3546298; doi:10.1007/s12265-012-9401-8)
Supplement: Supplementary file 1 — Electronic supplementary material (DOCX 326 kb) [file 12265_2012_9401_MOESM1_ESM.docx]

**ELECTRONIC SUPPLEMENTARY MATERIAL**

**Next generation diagnostics in inherited arrhythmia syndromes: a comparison of two approaches**

James S. Ware^1,2^, Shibu John^3^, Angharad M. Roberts^1^, Rachel Buchan^1^, Sungsam Gong^3^, Nicholas S. Peters^2^, David O. Robinson^4^, Anneke Lucassen^4,5^, Elijah R. Behr^6^, and Stuart A. Cook^1,3^

^1^MRC Clinical Sciences Centre, Imperial College London, UK

^2^National Heart and Lung Institute, Imperial College London, UK

^3^Cardiovascular Biomedical Research Unit, Royal Brompton & Harefield NHS Trust, UK

^4^Wessex Regional Genetics Laboratory, Salisbury NHS Foundation Trust, UK

^5^Faculty of Medicine, University of Southampton, UK

^6^St George’s University of London, UK

**Contents**

Supplementary Tables S1 – S5

Supplementary Figure S1**Supplementary Tables**

**Table S1.** Additional positive control variants not shared across platforms, for within-platform comparison of software performance.

| Variant type | Disease | Gene | Variant | Sample ID | Platform | PSS | GATK |
| --- | --- | --- | --- | --- | --- | --- | --- |
| SNP | CPVT | RYR2 | c.1244C>T | 80 | SOLiD | Yes | Yes |
| SNP | LQT | KCNQ1 | c.859G>A | 81 | SOLiD | No | No |
| SNP | LQT | KCNH2 | c.1744C>T | 81 | SOLiD | No | No |
| SNP | LQT | SCN5A | c.6016C>G | 81 | SOLiD | No | No |
| SNP | LQT | KCNH2 | c.3040C>T | 01 | SOLiD | No | No |
| SNP | ARVC | PKP2 | c.2489+1G>A | 47 | SOLiD | Yes | Yes |
| Indel | ARVC | PKP2 | c.2197_2202delinsG | 43 | SOLiD | Yes | No |
|  |  |  |  |  |  |  |  |
| SNP | LQT | KCNQ1 | c.1033G>C | 07 | 454 | Yes | Yes |
| SNP | LQT | KCNQ1 | c.1697C>T | 09 | 454 | Yes | Yes |
| SNP | LQT | KCNH2 | c.1277C>T | 34 | 454 | Yes | Yes |
| SNP | CPVT | RYR2 | c.14713T>C | 29 | 454 | Yes | No |
| SNP | CPVT | RYR2 | c.14713T>C | 30 | 454 | Yes | No |

Abbreviations: PSS = platform specific software, GATK = Genome Analysis Toolkit, Indel = Insertion or deletion, LQT = long QT syndrome, CPVT = catecholaminergic polymorphic VT, ARVC = arrhythmogenic right ventricular cardiomyopathy.

Reference sequences: KCNQ1= ENST00000155840, KCNH2=ENST00000262186, SCN5A=ENST00000333535, RYR2=ENST00000366574, PKP2=ENST00000070846.

**Table S2.** 49 genes sequenced using Hyb-SR approach. For each gene the percentage of bases callable is calculated as a median across the eight low multiplex samples.

| **Gene** | **Median bases callable (%)** |  | **Gene** | **Median bases callable (%)** |  | **Gene** | **Median bases callable (%)** |
| --- | --- | --- | --- | --- | --- | --- | --- |
| ABCC8 | 100.0 |  | GPD1L | 95.6 |  | PLN | 100.0 |
| ABCC9 | 100.0 |  | IL18 | 98.1 |  | RANGRF | 100.0 |
| ADRA2B | 99.2 |  | JUP | 100.0 |  | RNF207 | 83.7 |
| ADRB2 | 100.0 |  | KCNE1 | 100.0 |  | RYR2 | 99.8 |
| AKAP9 | 99.3 |  | KCNE2 | 100.0 |  | SCN1B | 73.0 |
| ANK2 | 99.5 |  | KCNE3 | 100.0 |  | SCN4B | 100.0 |
| ATP1B1 | 96.7 |  | KCNH2 | 76.2 |  | SCN5A | 99.9 |
| CACNA1C | 99.8 |  | KCNJ11 | 100.0 |  | SELP | 100.0 |
| CACNB2 | 97.3 |  | KCNJ2 | 100.0 |  | SERPINE1 | 100.0 |
| CASQ2 | 100.0 |  | KCNJ8 | 100.0 |  | SNTA1 | 79.7 |
| CAV3 | 100.0 |  | KCNQ1 | 83.4 |  | TGFB3 | 100.0 |
| DPP6 | 93.7 |  | KCNQ2 | 85.2 |  | TMEM43 | 100.0 |
| DSC2 | 99.4 |  | LIPC | 100.0 |  | TNNC1 | 100.0 |
| DSG2 | 98.7 |  | LITAF | 85.6 |  | TNNI3 | 100.0 |
| DSP | 99.2 |  | NDRG4 | 85.2 |  | TNNT2 | 99.0 |
| F7 | 93.2 |  | NOS1AP | 99.6 |  |  |  |
| GINS3 | 84.8 |  | PKP2 | 90.8 |  | **Median** | **99.8%** |

**Table S3.** Calculations of theoretical assay capacity for each approach, based on reported capture performance and sequencing output.

|  | **Hyb-SR** | **PCR-LR** |
| --- | --- | --- |
| Sequencing capacity (Mb) | 10,880^a^ | 40 |
| Usable sequencing capacity (Mb)^b^ | 3,264 | 40 |
| Target size (kb) | 448 | 42.0 |
| Intended sequencing depth | 200 | 20 |
| **Sample capacity** | **36**^c^ | **48** |

^a^128 million beads per quad, with 50 + 35bp paired reads.

^b^ Unmapped and off-target reads are to be expected with Hyb-SR, whereas the target enrichment specificity of PCR should approach 100%. This calculation anticipates that 60% of Hyb-SR map uniquely to the genome, with 50% from bait-targeted regions.

^c^ Pooling on the SOLiD platform was limited by the number of indexes available at the time of experimentation

**Table S4.** Sequencing metrics obtained with each strategy.

| **Total reads** | **No of Samples** | **Median reads per sample** | **Median forward read length (^a^bp)** | **Median reverse read length (bp)** | **Reads uniquely mapped (%)^b^** | **Mapped Reads on target (%)** | **Unique on-target reads (%)^c^** | **Enrichment Factor** |
| --- | --- | --- | --- | --- | --- | --- | --- | --- |
| **Hyb-SR low multiplex** | | | | | | | | |
| 198,713,052 | 8 | 22,190,617 | 47.1 | 33.1 | 68.8 | 25.4 | 16.4 | 5864 |
| **Hyb-SR high multiplex** | | | | | | | | |
| 164,958,748 | 28 | 5,589,971 | 48 | 30.7 | 64.5 | 14.8 | 49.2 | 3505 |
| **PCR-LR** |  |  |  |  |  |  |  |  |
| 90,206 | 45 | 2008 | 446.4 | NA | 76.6 | 89.2 | NA | 170,900 |
| **^a^**bp = base pairs  **^b^**reads mapping with an alignment quality score ≥8  **^c^**on target = protein coding bases. Each assay also aimed to capture some adjacent intronic sequence: 43% of Hyb-SR reads and 95% PCR-LR reads mapped to regions covered by baits/amplicons. | | | | | | | | |

**Table S5.** Contingency tables showing detection of positive control variants by variant-calling software, for each platform. GS junior data excludes indels, as these are not detected by GATK on this platform.

| **SOLiD** | Bioscope | |  | **GS junior** | AVA | |
| --- | --- | --- | --- | --- | --- | --- |
|  | Detected | Not detected |  |  | Detected | Not detected |
| GATK |  |  |  | GATK |  |  |
| Detected | 14 | 0 |  | Detected | 11 | 0 |
| Not detected | 6 | 6 |  | Not detected | 6 | 1 |
|  |  | p=0.031 |  |  |  | p=0.031 |

**Supplementary Figure**

**Figure S1.** A schematic representation of the laboratory workflow associated with each approach.
